# Phagocytosis by the retinal pigment epithelium: New insights into polarized cell mechanics

**DOI:** 10.1002/bies.202300197

**Published:** 2024-12-11

**Authors:** Ceniz Zihni

**Affiliations:** ^1^ Faculty of Health & Life Sciences Institute of Life Course and Medical Sciences University of Liverpool Liverpool UK

**Keywords:** cell mechanics, epithelium, epithelial polarity and adhesion, integrin adhesome, phagocytosis, retinal pigment epithelium

## Abstract

The retinal pigment epithelium (RPE) is a specialized epithelium at the back of the eye that carries out a variety of functions essential for visual health. Recent studies have advanced our molecular understanding of one of the major functions of the RPE; phagocytosis of spent photoreceptor outer segments (POS). Notably, a mechanical link, formed between apical integrins bound to extracellular POS and the intracellular actomyosin cytoskeleton, is proposed to drive the internalization of POS. The process may involve a “nibbling” action, as an initial step, to sever outer segment tips. These insights have led us to hypothesize an “integrin adhesome‐like” network, atypically assembled at apical membrane RPE‐POS contacts. I propose that this hypothetical network orchestrates the complex membrane remodeling events required for particle internalization. Therefore, its analysis and characterization will likely lead to a more comprehensive understanding of the molecular mechanisms that control POS phagocytosis.

## Graphical abstract

We propose the assembly of an atypical “integrin‐adhesome‐like” network, with a core integrin adhesive signaling patch (IASP), analogous to integrin adhesion complexes (IAC), at adhesion sites linking RPE apical integrin receptors, bound to photoreceptor outer segment (POS) tips damaged by light, to the actomyosin cytoskeleton. This network senses POS adhesion and responds by remodeling the membrane to “nibble” and internalize pieces of outer segment tips.

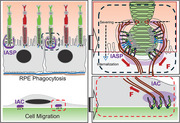

## INTRODUCTION

**FIGURE 1:**
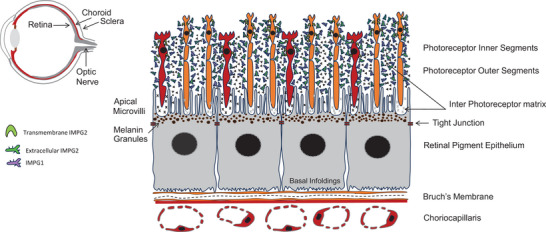
A schematic illustration of the polarized retinal pigment epithelium (RPE). The RPE is positioned at the boundary facing the neural retina comprised of rod and cone photoreceptor cells surrounded by interphotoreceptor matrix (IPM) at its apical microvilli covered membrane. The abundant IPM proteoglycans 1 and 2 (IMPG1 and IMPG2), are shown in purple and green, respectively. Transmembrane IMPG2 is reported to be localized strictly in the inner segment zone. Black and grey crosses represent distinct IPM constituents, presently poorly understood, at the cup rod and cone‐outer segment tip interface. At its basal side, the Bruch's membrane, a multilayered basement membrane, complexes the RPE to the vascular choriocapillaris.

The retina is a complex structure, located in the fundus of the eye, that plays an important role in vision. When light enters the eye, it is focused on the retina. Light signals are converted into electrical ones that then travel to the vision center of the brain via the optic pathway.^[^ ^1^ ^]^ Multiple cell types make up the multi‐layered structure of the retina, this review focuses on the retinal pigment epithelium (RPE)^[^ ^2^ ^]^ (Figure 1). The RPE is positioned at the boundary between the choroid and neural retina. On the microvilli‐covered apical/inner side, the RPE interfaces with photoreceptor cells (rods and cones) of the neural retina along with an interphotoreceptor matrix (IPM) in the avascular subretinal space (Figure 1). At its basal side, the RPE is complexed to the choroid, via the Bruch's membrane (BrM) (Figure 1), a five‐layer laminar extracellular matrix (ECM) sheet, rich in elastin and collagen.^[^ ^3^ ^]^ The RPE, together with the BrM and choroid on its basal side, collectively form the outer blood retinal barrier. The functions of the outer blood‐retinal barrier include the regulation of solute and nutrient passage to the sub‐retinal space from the choroid.^[^ ^4^, ^5^ ^]^

During retinal development, the neural retina secretes factors that regulate the assembly and maturation of RPE tight junctions (TJ), epithelial polarity, and tissue‐specific properties.^[^ ^6^ ^]^ The post‐mitotic RPE, typical of polarized epithelial cells, contains distinct apical and basolateral cell surface domains, defined by a discrete border formed by TJ ^[^ ^7^, ^8^, ^9^ ^]^ (Figure 1). In most epithelial monolayers, TJs regulate cell proliferation, and polarity, and form a barrier between neighboring cells by regulating paracellular transepithelial diffusion. The TJ transmembrane protein claudin‐19 plays a critical role in retinal differentiation, transepithelial permeability, and disease.^[^ ^10^ ^]^ RPE TJ and their claudin constituents are modulated by the neural retina and differ from other epithelia. The characteristics of RPE junctions, including the composition of claudins also show differences among species, suggesting variation in outer retina physiology amongst species.^[^ ^11^ ^]^

The apical‐basal polarized state of the RPE is essential to support photoreceptors on its apical side and the choroid on its basal side and to function as an outer blood‐retinal barrier.^[^ ^2^ ^]^ However, the mechanisms of RPE polarization are poorly understood. Protein sorting and endosomal compartment‐dependent transit, apical membrane interactions with the neural retina, TJs, and basolateral signaling with the BrM and choriocapillaris are each likely to play a role.^[^ ^12^, ^13^ ^]^ Disruption in RPE polarity is central to many degenerative retinal diseases. Therefore, an integrated understanding of these processes may provide insight into ocular disease.^[^ ^2^ ^]^

In the RPE, many proteins traditionally located on the basal aspect of epithelia are localized apically, including αvβ5 integrin.^[^ ^2^, ^14^ ^]^ The apical association of the RPE with an ECM, is also atypical when compared to the fluid‐filled lumina or cell sheets generally observed in other epithelial tissues. The IPM is a specialized ECM that surrounds photoreceptors. Its functions are thought to include the transportation of metabolites and nutrients between the RPE and photoreceptors, the presentation of growth factors, and communication between cells. The IPM has been reported to mediate retinal adhesion by forming a molecular bridge between the neural retina and RPE.^[^ ^15^, ^16^, ^17^ ^]^ Photoreceptors and the RPE contribute to the components of the IPM. Both generate a network of glycoproteins and proteoglycans that vary with region and sequester metabolites and growth factors.^[^ ^18^, ^19^, ^20^, ^21^, ^22^, ^23^ ^]^ Rods and cones are surrounded by different compositions of proteoglycans and glycoproteins.^[^ ^24^, ^25^ ^]^ This results in the formation of distinct sheaths that may facilitate the alignment of RPE microvilli and outer segments by providing mechanical support to both structures.^[^ ^23^ ^]^ Such an alignment may facilitate one of the major functions of the RPE, phagocytosis of spent photoreceptor outer segments (POS). The precise distribution of macromolecules in the IPM, or their mobility, is poorly understood. Recent work has provided new insight into two major proteoglycans of the IPM, known as IPM proteoglycan 1 and 2 (IMPG1 and IMPG2).^[^ ^20^, ^26^, ^27^, ^28^, ^29^ ^]^ Secreted IMPG1 is distributed throughout the IPM photoreceptor inner segment (IS) and POS zones, while a transmembrane IMPG2 is restricted to the IS zone (Figure 1). Transmembrane IMPG2 undergoes proteolysis, generating an extracellular form that relocates to around outer segments, a process dependent on IMPG1.^[^ ^16^ ^]^

## POS PHAGOCYTOSIS BY THE RPE

**FIGURE 2:**
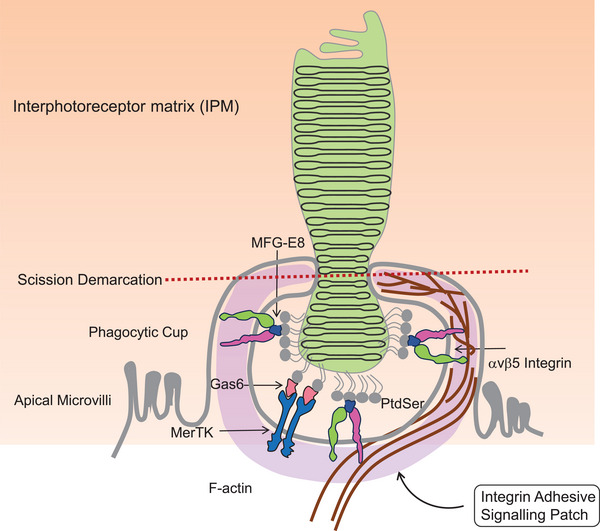
Extracellular interactions of RPE apical membrane with photoreceptor outer segment (POS) tips during phagocytosis. The tip of the POS accumulates phosphatidylserine (PtdSer) exposed on the outer leaflet of the cell (light exposure significantly increases PtdSer exposure on the outer leaflet). POS‐PtdSer tips binds to multiple receptors on the RPE apical membrane, including the αvβ5 integrin receptor via the ligand MFG‐E8 and the MerTK receptor via the ligand Gas6. Receptor binding of POS stimulates molecular pathways that become concentrated at the intracellular side of the apical membrane we term here an “integrin adhesive signaling patch” (IASP), depicted in purple. The IASP forms a physical link between the cytoplasmic segment of integrin and F‐actin to drive cytoskeleton‐dependent membrane remodeling to sever and internalize POS. This severing occurs at the scission demarcation region shown by the dotted line.

Phagocytosis of spent POS by the RPE, a circadian diurnal activity, has been investigated for more than 60 years. Previous work suggested that spent tips of POS are shed and then internalized.^[^ ^30^ ^]^ Recent work using a live‐cell imaging approach indicates that, on the contrary, OS tip scission occurs by the RPE.^[^ ^31^ ^]^ Demarcation of the site for scission may be due to the distribution of the phospholipid, PtdSer^[^ ^32^ ^]^ (Figure 2). Although the size of the ingested tip is consistent with the term “phagocytosis,” this term usually refers to the internalization of an entire particle or cell. A process described as trogocytosis has been proposed, where one cell “nibbles” another cell.^[^ ^31^ ^]^

The mechanism of POS phagocytosis is not well understood but requires the binding of apical αvβ5 integrin receptors of the RPE to the milk fat globule‐EGF 8 (MFG‐E8) protein.^[^ ^14^, ^33^ ^]^ Mechanically stable association of the RPE to IPM components, and spent POS during diurnal phagocytosis may both require αvβ5 integrin receptor function.^[^ ^30^, ^34^ ^]^ It has been postulated that a varying availability of MFG‐E8 in the IPM may rhythmically affect αvβ5 integrin receptor activation during diurnal POS phagocytosis.^[^ ^35^ ^]^ In support of this model, a lack of αvβ5 integrin in mice causes weakened retinal adhesion and asynchronous phagocytosis.^[^ ^36^ ^]^ The activities of FAK and MerTK (discussed further below) are both required for POS internalization.^[^ ^37^, ^38^, ^39^ ^]^ TJs have also been implicated in the later stage of phagocytosis via an undefined mechanism indicating a dynamic interplay between the different cell adhesion networks. Knockdown of claudin‐19 was found to result in reduced POS degradation, without affecting internalization.^[^ ^40^ ^]^ It should be noted that other forms of RPE phagocytosis, such as internalization of latex beads do not involve αvβ5 integrin receptors, and represent a distinct mechanism.^[^ ^14^ ^]^ Since the present hypothesis focuses on a proposed apical αvβ5 integrin adhesome‐like network, that is, assembled during POS engulfment, this article will only consider this process.

### Proposed phagocytic RPE “integrin adhesome‐like” network

Integrins are transmembrane proteins that link the ECM to the cell cytoskeleton.^[^ ^41^, ^42^, ^43^ ^]^ Since their initial identification in the 1980s, a large body of work has established integrins as fundamental receptors of cell adhesion. Integrins mediate cell and tissue function in a wide range of processes in health, including cell migration, proliferation, and differentiation. Integrins, therefore, play a key role in how individual cells assemble into functional tissues and organs, and diseases such as cancer.^[^ ^44^, ^45^, ^46^, ^47^, ^48^ ^]^ In these processes integrin receptors are direct mediators of adhesion with the ECM or with neighboring cells that contain counter receptors.^[^ ^43^ ^]^ Focal adhesions (FAs) are a common form of integrin adhesions that have been extensively studied using in vitro cell models. At FAs a complex network of proteins referred to as the “integrin adhesome” links the cytoplasmic side of integrins with the cytoskeleton.^[^ ^49^, ^50^, ^51^, ^52^, ^53^ ^]^

A large body of work and analysis of the integrin adhesome has identified a set of proteins, within a core group of integrin adhesion complex (IAC) proteins, termed consensus adhesome proteins. These components, including focal adhesion kinase (FAK), paxillin, talin, and vinculin, have been commonly identified in IAC isolate preparations. These consensus proteins, that contribute to the “molecular clutch” (see below), were found to recruit a wide variety of non‐consensus adhesome proteins of the IAC.^[^ ^52^, ^54^ ^]^ This indicates that they may form diverse networks with various non‐consensus adhesome components during different processes. Indeed, interactions between different integrin adhesome components are thought to modulate physical integration between the actin cytoskeleton and the ECM‐bound cell. This leads to tissues assembled with different mechanical properties and structures. In addition to its integrative or “scaffold” role, such interactions play a “signaling” role. This allows cells to sense and respond to the mechanical and chemical properties of the extracellular environment. The cell responds by activating signaling networks that regulate the structure of the cell along with its behavior, dynamics, and fate.^[^ ^55^, ^56^, ^57^, ^58^ ^]^ Extensive studies have shown that FAs transmit traction force against the ECM. This force is generated by the activity of the integrin adhesome‐associated actomyosin cytoskeleton. Through the many signaling pathways integrated with the integrin adhesome these structures can play important roles in cell migration, the sensing of substrate stiffness, and ECM remodeling.^[^ ^59^, ^60^, ^61^ ^]^

The association of the apical RPE with the IPM and POS (Figure 2), via αvβ5 integrin,^[^ ^36^ ^]^ and its assembly of molecular clutch components during phagocytosis ^[^ ^62^ ^]^ (see below), leads us to propose parallels with IACs. These distinct apical features of the RPE could hypothetically form the basis of an integrin adhesome‐like network, atypically, at its apical side, under particular conditions. For instance, apical RPE IAC‐like networks may be required during contacts made with POS discs. Such networks would likely sense POS adhesion and provide a cellular response that involves complex membrane remodeling and the generation of force to drive “nibbling” and internalization.

### MerTK as a co‐activator of an integrin adhesome‐like network during POS phagocytosis

In the RPE, integrin‐dependent adhesion to POS, requires cooperation with, the receptor tyrosine kinase, MerTK for the internalization of POS. The importance of MerTK in phagocytosis is evident since inherited inactivating variants of MerTK are found in individuals suffering from retinitis pigmentosa. These mutations lead to an inhibition of phagocytosis, retinal degeneration, and vision loss.^[^ ^63^, ^64^ ^]^

Previous studies collectively suggest the involvement of a complex network of downstream effector mechanisms of MerTK during POS phagocytosis.^[^ ^65^ ^]^ Once engaged, MerTK may promote the recruitment of proteins necessary for αvβ5 integrin activation (see below).^[^ ^65^ ^]^ MerTK‐mediated phagocytosis has been reported to involve focal adhesion kinase (FAK).^[^ ^37^, ^65^, ^66^ ^]^ Phagocytosis driven by MerTK also involves PtdIns (4,5) P2 (PIP2), that is, cleaved to generate Ins3P, associated with integrin and calcium signaling, and diacylglycerol (DAG), that is, associated with Rap activation.^[^ ^67^, ^68^, ^69^ ^]^ The proposed trogocytosis‐like mechanism of POS engulfment may use a process of POS ensheathment, that involves the production of protrusions larger than microvilli. Ultrastructural analysis indicates that MerTK ligands PROS1 and GAS6 stimulate the ensheathment of POS which is required to fragment POS prior to internalization.^[^ ^70^ ^]^ MerTK engagement promotes multimerization and kinase domain‐dependent trans‐auto‐phosphorylation. This results in the recruitment of signaling proteins inducing actin polymerization and the extension of pseudopods that may be dependent on Rac (Reviewed by Kwon and Freeman^[^ ^65^ ^]^). A more recent study indicates the involvement of a related small GTPase, Cdc42 in actin polymerization,^[^ ^62^ ^]^ described in detail below. MerTK‐activated actin branching‐driven membrane protrusions are accompanied by the removal of highly bundled actin cables at the submembrane cortex.^[^ ^62^, ^65^ ^]^ This process is also controlled by MerTK, which recruits and activates PI3K which leads to the recruitment of Rho GAPs to this region.^[^ ^65^ ^]^ In addition to promoting POS engulfment MerTK is suggested to limit POS‐αvβ5 integrin binding via a negative feedback loop,^[^ ^39^ ^]^ that contributes to the rhythmic control of phagocytosis. Other studies suggest the cell surface cleavage of MerTK ^[^ ^71^ ^]^ also contributes to this process. A recent study suggests a role for MerTK‐activated myosin II ^[^ ^62^ ^]^ in driving POS engulfment, supporting previous work.^[^ ^72^ ^]^ This would provide a possible explanation for how the proposed scission of POS may be achieved (discussed below).

### Myosin II in phagocytosis

The role of myosin‐II in general phagocytosis is well studied but remains controversial. For instance, in macrophages, complement receptor‐mediated phagocytosis was found to be myosin‐II independent.^[^ ^73^ ^]^ Other studies using macrophages, during FcR‐mediated internalization, suggested myosin‐II may not play a direct role in cup formation.^[^ ^74^, ^75^ ^]^ Myosin‐II has been suggested, hypothetically, to play a role in particle internalization since it increases cortical tension, a property that propels the inward movement of a particle.^[^ ^76^, ^77^ ^]^ However, the dissection of the specific role of myosin‐II, if any, has been difficult. Furthermore, it has been argued that in some processes, myosin function may be independent of its motor activity,^[^ ^78^ ^]^ although such processes still probably result from contractility and tension generation.^[^ ^79^ ^]^ A recent study of macrophages using advanced imaging techniques, has overcome some of these problems and shed more light on a possible role for myosin‐II in Fc receptor‐mediated phagocytosis.^[^ ^80^ ^]^ This study indicates spatially localized forces, largely driven by ARP2/3‐mediated assembly, of discrete actin protrusions throughout phagocytosis. Contractile myosin‐II activity contributes to late‐stage phagocytic force generation and progression.^[^ ^80^ ^]^ This is in agreement with the hypothesized myosin‐II driven inwards movement of particles.^[^ ^77^ ^]^ Another recent study of Fc‐R mediated phagocytosis by macrophages supports a role for myosin‐II‐associated signaling in late‐stage phagocytosis.^[^ ^62^ ^]^ These studies, therefore, collectively indicate that the involvement of myosin‐II in phagocytosis may depend on the receptor type.

**FIGURE 3:**
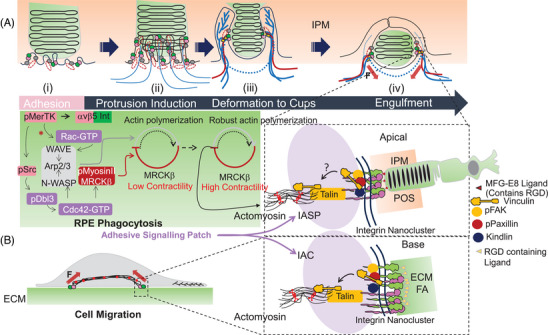
Proposed core components of the integrin adhesome‐like network assembled at RPE‐POS integrin adhesive signaling patches. (A) MerTK‐activated F‐actin polymerization and MRCKβ‐myosin‐II activity progresses from low levels during initial adhesion to high levels, during cup formation and initiation of particle engulfment. Note that initial protrusion formation is likely to involve actin branching. The asterisk signifies multiple mechanisms of MerTK‐Rac‐1 activation resulting in actin polymerization and branching, reviewed by Kwon and Freeman.^[^ ^65^ ^]^ MerTK signaling is also implicated in the “inside‐out” activation of αvβ5 integrin via talin.^[^ ^65^ ^]^ Continued F‐actin polymerization, and myosin‐II activity stimulate the linkage of the actomyosin cytoskeleton to integrin receptors via the mechanotransducer talin and reinforcement of the mechanical bridge via increased vinculin recruitment to accommodate increased force transmission during POS internalization. (B) The core signaling network assembled at POS‐RPE integrin adhesive signaling patches (IASP) shows similarity to the well‐established core integrin adhesion complex (IAC) formed at focal adhesions of migrating cells. Adhesions are associated initially with pushing branched F‐actin at peripheral nascent adhesions at the lamellipodial edge of migrating cells. Then they become associated with pulling contractile bundles of linear filaments at FAs.^[^ ^121^ ^]^ Both are depicted in purple zones. Apical POS‐RPE integrin adhesive signaling patches may promote similar actomyosin driven pulling forces to FAs on basal ECM, to internalize POS. Note that the MFG‐E8 ligand contains an integrin‐binding RGD motif.

In the RPE, a recent study indicates that myosin‐II drives distinct morphological membrane transformations ranging from cup morphogenesis to internalization of POS (Figure 3A).^[^ ^62^ ^]^ This work characterized an effector mechanism of the MerTK receptor, which stimulates phosphorylation and activation of the apical Cdc42 GEF Dbl3.^[^ ^81^ ^]^ Dbl3 activates Cdc42 at nascent POS‐membrane contacts to drive myosin‐II dependent phagocytosis via MRCKβ (Figure 3A).^[^ ^62^ ^]^ MRCKs are a family of kinases, related to the Rho A effector ROCK, displaying diverse functions at the polarized cortex of various cell and tissue remodeling processes.^[^ ^82^, ^83^, ^84^ ^]^ Db3 has previously been shown to function as an apical determinant of polarity in mammalian cells.^[^ ^81^ ^]^ Its apical localization to the membrane is partly dependent on its interaction with the brush border protein ezrin, which promotes the morphogenesis of apical microvilli in RPE.^[^ ^85^ ^]^ A recent study indicates that O‐GlcNAcylation‐dependent ezrin localization to the apical RPE cortex is important for phagocytosis and retinal integrity.^[^ ^86^ ^]^ POS‐membrane adhesion results in a rapid enrichment of the receptors αvβ5 integrin and MerTK at nascent POS‐membrane adhesion sites.^[^ ^62^ ^]^ Referred to from here on as “integrin adhesive signaling patches” (IASPs), these sites are enriched with Cdc42 signaling machinery.

Cdc42 stimulates actin polymerization, probably via ARP2/3, to generate membrane protrusions.^[^ ^62^ ^]^ Since previous work indicates that Rac may also play an important role in ARP2/3 directed actin polymerization, activated by MerTK (reviewed by Kwon and Freeman ^[^ ^65^ ^]^), this would indicate a potential cooperation between the two pathways (Figure 3A). In FcR‐mediated macrophage phagocytosis Rac and Cdc42 have been reported to display localization and morphogenetic profiles that are spatiotemporally coordinated (Hoppe and Swanson, 2004). MRCKβ regulated actomyosin dynamics then proceeds to control the maturation of protrusions into cups that wrap around POS and internalize it.^[^ ^62^ ^]^ The results from this study indicate that myosin‐II dependent actomyosin contractility controls cup shaping by providing a limiting step to actin polymerization. These findings are in agreement with a previously proposed model whereby an interdependence of contractility and actin polymerization creates appropriate conditions for complex morphodynamic processes.^[^ ^87^ ^]^

In addition to myosin‐II, other myosins have been implicated in the RPE phagocytosis of POS. Myosin‐VIIa functions in late‐stage POS phagocytosis by the RPE.^[^ ^88^, ^89^ ^]^ Following ingestion of POS disc membranes, the RPE functions to degrade phagosomes. As phagosomes moved away from the apical region of the RPE, they were reported to associate with myosin‐VIIa, followed by kinesin‐1.^[^ ^89^ ^]^ These results collectively suggest that myosin‐II and myosin‐VIIa may function sequentially to engulf, then transport, phagosomes in an apical to basal direction within the cell. More recently, myosin‐VI has also been implicated in POS internalization.^[^ ^90^ ^]^ The authors suggest that myosin‐VI may be involved in the scission step of RPE phagocytosis, with the cytosolic protein optineurin.^[^ ^90^ ^]^ Optineurin interacts with myosin‐VI, amongst other proteins, and is involved in various membrane trafficking and signaling functions.^[^ ^91^, ^92^ ^]^ Myosin‐VI is an atypical minus end‐directed motor involved in various trafficking pathways, including endocytosis.^[^ ^93^ ^]^ One of the major functions of Myosin‐VI is the maintenance of microvilli structure, composition, and membrane trafficking in mammalian epithelial cells.^[^ ^94^ ^]^ Therefore, it is unclear at present, whether optineurin and myosin‐VI, if functioning together in this process, play a direct or indirect role in POS internalization.

### “Molecular clutch” proteins in RPE phagocytosis

The integrin adhesome includes a mechanically responsive assembly of “core” molecules termed the “molecular clutch,” that plays an important part in the organization of FAs (Figure 3B).^[^ ^45^ ^]^ The molecular clutch was originally proposed by Tim Mitchison and Marc Kirschner in 1988 to explain the way tension is exerted on a substrate by actin retrograde flow.^[^ ^95^ ^]^ The term refers to the mechanical linkage formed by dynamic interactions between activated integrins bound to ECM and the actomyosin cytoskeleton that generates force.

Integrin activation is an important mechanism whereby cells can regulate integrin function by modulating its affinity to ECM ligands, and bi‐directional signaling to facilitate mechano‐sensing and mechano‐transduction.^[^ ^53^ ^]^ Integrins can exist in different states of ligand affinity. The low ligand affinity state is where the extracellular head is bent, whereas in the high ligand affinity “active” state the extracellular head domain is fully extended. The different conformations adopted by integrins are in a dynamic equilibrium that modulates their affinity for ECM.^[^ ^96^, ^97^, ^98^ ^]^ Integrins may be regulated from the inside, that is, a process dependent on the binding of an inhibitor or the activator talin to the cytoplasmic β‐tail.^[^ ^53^, ^99^, ^100^, ^101^, ^102^, ^103^, ^104^ ^]^ The signaling between MerTK and integrins are not mutually exclusive ^[^ ^105^ ^]^ and MerTK can initiate inside‐out activation of integrins, including αvβ5 integrin. MerTK receptor engagement initiates a cascade of recruitment initially involving SH2‐containing proteins such as Vav, Grb2, and Crk adaptors and PLCγ to phosphorylated tyrosine residues on the MerTK cytoplasmic tails. This, in turn, favors recruitment of Rap GTPases that can lead to the recruitment of talin (reviewed by Kwon and Freeman ^[^ ^65^ ^]^). Other factors that influence the conformation of integrins include contractility driven mechanical forces applied to integrins, and ECM tension.^[^ ^57^, ^106^, ^107^ ^]^

In migratory cells, nascent adhesions form when nanoclusters of approximately 50 activated integrins associate with both the ECM and core adhesome proteins. FAK, paxillin, talin, and kindlin associate with integrins via their cytoplasmic tails to form approximately 100 nm puncta.^[^ ^53^ ^]^ The maturation of nascent adhesions to focal adhesions (FAs) involves nanocluster maturation. This process requires Src‐family kinase (SFK) and RhoA signaling that initiates actin polymerization via formin and likely ARP2/3, and activation of actomyosin contractility, respectively. Another prerequisite for nanocluster maturation is talin association with actin filaments undergoing retrograde flow. This engagement leads to talin being mechanically activated, partially unfolding, and the recruitment of further signaling and adaptor proteins, including activated vinculin.^[^ ^108^ ^]^ Recruitment of activated vinculin enables the formation of additional crosslinks between actin and talin to promote high ECM traction force and the stabilization of the active integrins.^[^ ^53^, ^57^, ^106^, ^107^ ^]^

It has been suggested that talin forms a “chassis”‐like role in its relationship with vinculin which acts as a regulatable “moving part.” This is thought to modulate mechanical, structural, and signaling aspects of IACs.^[^ ^53^ ^]^ Studies using super‐resolution microscopy (SRM) have revealed a multilaminar structural arrangement of the vertical axis of FAs.^[^ ^109^, ^110^, ^111^, ^112^ ^]^ The arrangement comprises three distinct layers: the actin regulatory layer (ARL), the integrin signaling layer (ISL), and the middle force transduction layer (FTL). The normally autoinhibited vinculin possesses multiple binding sites for different proteins of the IAC.^[^ ^113^ ^]^ Auto‐inhibited vinculin can localize to the ISL due to interaction with phospho‐paxillin (Y31 and Y118).^[^ ^114^ ^]^ The force exerted on talin to expose vinculin‐binding sites is sufficient to activate vinculin at FAs, and binding to talin promotes localization to the FTL.^[^ ^114^, ^115^ ^]^ As the IAC matures, vinculin can progressively re‐localize to the ARL ^[^ ^114^ ^]^ (Figure 3B), hence its comparison to a “moving” component.^[^ ^53^ ^]^

MRCKβ‐mediated myosin‐II activity, turned on by MerTK engagement, in the RPE drives POS engulfment via contractile force‐dependent pulling into the cell.^[^ ^62^ ^]^ MRCK‐driven actomyosin contractility activates FAK, stimulates integrin receptor clustering and assembly of talin and vinculin components of the molecular clutch at IASPs (Figure 3A). The utilization of the molecular clutch during POS engulfment by the RPE was first postulated by Kwon and Freeman.^[^ ^65^ ^]^ Myosin‐II‐dependent FAK activation in the RPE may function to promote the recruitment of mechano‐transducers during phagocytosis, similar to mesenchymal migration. Here, FAK activation, dependent on myosinII, mediates paxillin phosphorylation. This leads to vinculin binding to talin and actin and hence reinforces the molecular clutch between the cytoskeleton and ECM.^[^ ^116^, ^117^, ^118^, ^119^, ^120^ ^]^ Whether a similar multi‐laminar architecture, or the multimode “chassis” and “moving part” interplay of talin and vinculin, observed at IACs, exists at RPE IASPs remains to be determined.

## SUMMARY AND TESTING THE HYPOTHESIS

It is proposed that the constituent proteins identified at apical RPE IASPs, form an integrin “adhesome‐like” network. Rather than the IAC linking the ECM to actomyosin at FAs in migrating cells, for instance, the apical IASP network links extracellular POS to actomyosin during phagocytosis (Figure 3). In a process described as trogocytosis, the RPE cell “nibbles” at the POS tips. Actin filaments were enriched at sites of POS scission,^[^ ^31^ ^]^ which may play an important role in regulating the size of the POS tip internalized and its time course. Other recent work reported actin filaments enriched around POS at cups in vitro. This was accompanied by an increase in actomyosin contractility and the assembly of molecular clutch components.^[^ ^62^ ^]^ These findings could potentially explain how forces required for POS scission might be generated. In agreement with this hypothesis, Voreselen et al. demonstrated actomyosin contractility generated during particle internalization was sufficient to deform soft targets.^[^ ^80^ ^]^ Previously, Swanson and colleagues demonstrated a “purse‐string” like mechanism used to constrict phagocytic targets that were sufficient to sever through a red blood cell.^[^ ^121^ ^]^ It is, therefore, plausible that the RPE uses actomyosin contractility, associated with a complex apical integrin adhesome‐like signaling network, to orchestrate the squeezing and pulling of POS tips. This could effectively result in their “nibbling” during the engulfment process with the sophisticated force feedback machinery of the IASP network regulating this process.

The present hypothesis may be initially tested using a method recently described to study the role, nature, and dynamics of actin polymerization and myosin‐II‐contractility‐generated mechanical forces during macrophage phagocytosis.^[^ ^80^ ^]^ In this study Vorselen et al. combined lattice light‐sheet microscopy (LLSM) and microparticle traction force microscopy (MP‐TFM) to demonstrate assembly of discrete actin protrusions. These structures, containing myosin‐1e and myosin‐1f, appeared to connect with each other in a ring‐like organization and served as “teeth.” The ring‐like structures then went on to undergo myosin‐II dependent contractile activity. This contributed to the “jaw” of the process of late‐stage force generation and progression during phagocytic cup closure. The group observed partial “eating” attempts reminiscent of trogocytosis.^[^ ^80^ ^]^ Therefore, this approach could be used to test the importance of actin polymerization and actomyosin contractions in POS “nibbling” and internalization by the RPE. The following experimental approaches may be employed.

### Analysis of RPE cup cytoskeletal dynamics and phagocytic forces

Lattice light‐sheet microscopy (LLS) can be used for high‐speed volumetric real‐time imaging of cytoskeletal dynamics and phagocytic forces during POS internalization in a 3D environment. Porcine RPE cells, isolated as previously described,^[^ ^62^ ^]^ could be probed with SiR F‐actin to visualize filamentous actin.^[^ ^122^ ^]^ POS may be visualized by labeling with FITC.^[^ ^62^ ^]^ 3D shape reconstructions of POS fed to RPE would enable the real‐time analysis of POS deformations as a direct readout of phagocytic forces. A similar approach was described recently, using macrophages fed with microparticles.^[^ ^80^ ^]^ To analyze the role of the actin cytoskeleton in force generation in more detail microparticle traction force microscopy (MP‐TFM) may be applied to fixed cells, fed with POS‐FITC, and stained for F‐actin. The engulfment stage can be determined by immunostaining of the exposed particle surface. Confocal z‐stacks of RPE phagocytic cups could be used to create 3D POS shape reconstructions at high‐resolution accuracy. In addition, confocal z‐stacks would enable the analysis of traction forces to infer the contributions of normal and shear forces to mechanical interaction during POS engulfment.^[^ ^123^ ^]^

### Role of actin polymerization and myosin‐II contractility on POS “nibbling” and engulfment

To dissect the roles of ARP2/3‐mediated actin polymerization and myosin‐II mediated contractility during POS phagocytosis, an inhibitory approach could be used.^[^ ^80^ ^]^ By combining target deformation analysis and force calculations ^[^ ^80^ ^]^ the role of ARP2/3 and myosin II could be studied during POS engulfment by RPE cups. ARP2/3‐actin polymerization drives force generation throughout Fc‐receptor mediated macrophage phagocytosis.^[^ ^80^ ^]^ Myosin‐II, on the other hand, functions during cup closure. Using MP‐TFM Vorselen et al. observed discrete spots of inward deformation dependent on ARP2/3, representing “tooth‐like” projections indenting the target surface along the internal rim of the phagocytic cup.^[^ ^80^, ^124^ ^]^ Using LLSM the group tracked the dynamics of branched actin teeth, containing actin‐binding proteins, cortactin, and cofilin. A forward movement was observed over the surface of the target during phagocytosis. Myosin‐II appeared behind the actin teeth along with paxillin and vinculin, possibly participating in the interconnection of the teeth. Myosin‐II localized at sites of target constriction where extreme deformation was observed, during completion of cup closure. Almost 40% of particles were deformed but not internalized indicating attempts at trogocytosis.

To analyze the localization of myosin‐II during POS deformation as a process of “nibbling,” RPE cells could be transfected with GFP‐tagged myosin light chain, using mRNA to achieve high transfection efficiency.^[^ ^125^ ^]^ Myosin‐II promoted the disassembly of actin at the base of the macrophage cup as the particle internalized.^[^ ^80^ ^]^ This draws parallels to myosin‐II dependent disassembly of F‐actin at the rear of a motile cell.^[^ ^80^, ^126^ ^]^ In the RPE it is unclear whether myosin‐II plays a similar role since the inhibition of RhoA signaling has been implicated in the disassembly of linear actin at the base of cups.^[^ ^65^ ^]^ It is possible RhoA may be tightly regulated to control the differential activities of ROCK‐I and ROCK‐II ^[^ ^127^ ^]^ during linear actin disassembly. MRCKβ‐activated Myosin‐II was found to limit actin polymerization to control RPE cup remodeling to internalize POS.^[^ ^62^ ^]^ Therefore, this approach is likely to provide further insight into the remodeling of the actin cytoskeleton at RPE cups during POS engulfment. In addition, inhibitory effects on Myosins‐VIIa and ‐VI could be examined to determine the potential spatiotemporal interplay between them during engulfment. Studies suggest Rac and Cdc42 signaling may converge to control ARP2/3 dependent actin polymerization during RPE phagocytosis ^[^ ^62^, ^65^ ^]^ (Figure 3). A loss‐of‐function approach may be applied to both small GTPases and their effectors. This could potentially dissect the precise interplay between the two processes and their effects on protrusion induction and force generation during POS engulfment.

Methods used to characterize the integrin adhesion complexes at focal adhesions could be applied to IASPs at RPE integrin‐POS adhesions. Such studies would enable the characterization of proposed integrin adhesome‐like networks during different stages of POS engulfment. These data could then be correlated to actomyosin dynamics and localized force generation results determined in the previous approaches. The following methods may be employed to determine the constituents, their potential interactions, and hierarchy at RPE IASPs during phagocytosis.

### Analysis of apical Integrin‐adhesome like networks during POS Phagocytosis

The development of methods to isolate and determine the chemical composition of the IAC^[^ ^52^ ^]^ may, in principle, be applied to study the RPE IASP, during POS phagocytosis. Previous work has identified adhesome proteins in integrin‐containing ventral membrane preparations. IAC complexes were enriched by removing the cell body and cytoplasmic proteins. Chemical crosslinkers were used to stabilize IACs.^[^ ^52^ ^]^ Theoretically, similar principles, may be applied and customized to study POS bound IASPs, specifically, during different stages of phagocytosis. Mass spectrometry and proteomics could then be used to determine IASP composition, as previously applied to the IAC of FAs.^[^ ^52^ ^]^ A complementary, or alternative, approach which does not require the isolation of intact IACs is based on proximity‐dependent biotin identification (BioID). This method dissects different integrins by labeling specific integrin interaction partners in live cells.^[^ ^128^, ^129^ ^]^ The protein of interest is fused to a promiscuous biotin ligase enzyme tag. When expressed in cells the fused protein permanently labels any protein within a 10 nm radius allowing mass spectrometric identification. This approach provides protein‐protein interaction data, and potentially information on more transient and weaker interactions.

### Analysis of spatiotemporal interactions and protein architecture during phagocytosis

Various approaches may be employed to study protein interactions and post‐translational modifications at IASPs spatiotemporally. For example, the in‐situ proximity ligation assay (PLA) can detect protein‐protein interactions and phosphorylation prepared for immunofluorescence microscopy analysis.^[^ ^130^ ^]^ Two targets are detected using primary antibodies bound to a pair of secondary antibodies. These antibodies are conjugated to complementary oligonucleotides that act as the PLA probes. If the probes are in close proximity a signal is generated and visualized in the form of a fluorescent spot. Using appropriate image analysis software and microscopy image overlay, the PLA signals can be quantified and assigned to a subcellular location, respectively. In addition, a combination of various biochemical, proteomics and computational methods may be employed, as used to investigate IACs.^[^ ^131^, ^132^, ^133^ ^]^

The deployment of super‐resolution microscopy over the last 20 years has facilitated the analysis of the internal 3D molecular organization of the IAC of both FAs and podosomes at nanoscale levels.^[53,^ ^109^, ^110^, ^111^, ^134^ ^]^ This approach may potentially be used to study the 3D organization of IASPs from initial nascent adhesion to POS, to maturation of IASPs. It would be interesting to determine whether IASPs also display a multilaminar architecture and whether the proposed molecular clutch displays a similar “chassis and moving part” mechanism as suggested for the IAC at FAs.^[^ ^53^ ^]^

## CONCLUSIONS

Characterizing the proposed RPE integrin adhesome‐like network during phagocytosis is likely to provide a more comprehensive understanding of the mechanisms that orchestrate POS internalization. Furthermore, such an analysis will potentially provide a better understanding of the mechanisms that control retinal integrity and broader functions of the RPE. In addition to contacts made with POS, it is plausible that changes in the biochemical and/or mechanical properties of the IPM may also be sensed by transiently formed integrin adhesome‐like networks. This would enable the RPE to elicit a response, during various functions within the RPE‐neural retina region, to facilitate a given process. Studies have shown that the biochemical composition and physical properties of the ECM are important regulators of cell behavior.^[^ ^135^ ^]^ The most extensively characterized property of the ECM is its elasticity. This can be defined by a material's ability to undergo a non‐permanent type of deformation. Studies using primate retina indicate the IPM may possess very elastic properties. ^[^ ^17^ ^]^ The effect of changes in the chemical and mechanical properties of the IPM on different RPE functions, including phagocytosis, warrants investigation. This is especially important considering its potential impact on the retina in heath and disease.^[^ ^15^, ^16^ ^]^ For example, recent work suggests mutations in IMPG1 and IMPG2, which result in inhibition of proteolysis, are associated with human vision disease. The co‐dependency of IMPGs on their distribution implicates mutations in either proteoglycan in the disruption of retinal function and progression of retinal disease.^[^ ^16^ ^]^ Another important group of elements involved in the tight regulation of ECM remodeling over time are matrix metalloproteinases (MMPs).^[^ ^136^ ^]^ As in other systems MMPs in the IPM are thought to play a vital role in the turnover of components containing protein and their imbalance may be associated with retinal diseases such as AMD.^[^ ^137^ ^]^

## AUTHOR CONTRIBUTIONS

Ceniz Zihni wrote the manuscript, drafted the figures and graphical abstract.

## CONFLICT OF INTEREST STATEMENT

The authors declare no conflicts of interest.

## Data Availability

Data sharing is not applicable to this article since datasets were not generated or analyzed during the present hypotheses study.
